# Comparative Insight upon Chitosan Solution and Chitosan Nanoparticles Application on the Phenolic Content, Antioxidant and Antimicrobial Activities of Individual Grape Components of Sousão Variety

**DOI:** 10.3390/antiox9020178

**Published:** 2020-02-21

**Authors:** Vanessa Silva, Rupesh Kumar Singh, Nelson Gomes, Bruno Gonçalves Soares, Adriana Silva, Virgílio Falco, Rosa Capita, Carlos Alonso-Calleja, José Eduardo Pereira, Joana S. Amaral, Gilberto Igrejas, Patrícia Poeta

**Affiliations:** 1 Microbiology and Antibiotic Resistance Team (MicroART), Department of Veterinary Sciences, University of Trás-os-Montes and Alto Douro (UTAD), 5000-801 Vila Real, Portugal; vanessasilva@utad.pt (V.S.); adrianaa.silva95@gmail.com (A.S.); jeduardo@utad.pt (J.E.P.); 2 Department of Genetics and Biotechnology, Functional Genomics and Proteomics’ Unit, University of Trás-os-Montes and Alto Douro, 5000-801 Vila Real, Portugal; nelsondpg710@gmail.com (N.G.); gigrejas@utad.pt (G.I.); 3 Functional Genomics and Proteomics Unit, University of Tras-os-Montes and Alto Douro (UTAD), 5000-801 Vila Real, Portugal; 4 Laboratório Associado for Green Chemistry (LAQV-REQUIMTE), University NOVA of Lisboa, Lisboa, 2829-516 Caparica, Portugal; 5 Centro de Química-Vila Real (CQ-VR), University of Trás-os-Montes and Alto Douro, 5000-801 Vila Real, Portugal; rupeshbio702@gmail.com (R.K.S.); vfalco@utad.pt (V.F.); 6 CoLAB Vines&Wines—National Collaborative Laboratory for the Portuguese Wine Sector, Associação para o Desenvolvimento da Viticultura Duriense (ADVID), 5000-801 Vila Real, Portugal; bruno.soares@advid.pt; 7 Department of Food Hygiene and Technology, Veterinary Faculty, University of León, E-24071 León, Spain; rosa.capita@unileon.es (R.C.); carlos.alonso.calleja@unileon.es (C.A.-C.); 8 Institute of Food Science and Technology, University of León, Av. Facultad de Vetrinaria, 25, 24004 León, Spain; 9 CECAV, 5000-801 Vila Real, Portugal; 10 Centro de Investigação de Montanha (CIMO), Polytechnic Institute of Bragança, Campus de Santa Apolónia, 5300-253 Bragança, Portugal; jamaral@ipb.pt; 11 REQUIMTE-LAQV, Faculty of Pharmacy, University of Porto, Rua de Jorge Viterbo Ferreira 228, 4050-313 Porto, Portugal

**Keywords:** chitosan, chitosan nanoparticles, grapevines, by-products, antioxidant, antimicrobial, phenolics

## Abstract

Chitosan, a natural polysaccharide, has been previously proposed as an elicitor in plants to prevent pathogen infections. The present study aimed to analyze the effect of chitosan solution and chitosan nanoparticles treatment applied on the grapevine variety Sousão with respect to the phenolic composition, antioxidant potential and antibacterial activity of its individual grape components. Grapevine plants of selected lines were sprayed with chitosan solution and chitosan nanoparticles, and ethanolic extracts of stems, seeds and skins were prepared from grapevines treated and not treated with chitosan. Total phenolic, anthocyanin and tannin contents were studied, and the identification of the individual phenolic compounds was performed by HPLC-DAD. The antimicrobial susceptibility method was performed using the Kirby-Bauer disc diffusion method against multidrug-resistant bacteria. Overall, there was small increase in the concentration of phenolic compounds, antioxidant and antimicrobial activities in grape components treated with chitosan solution. Seed extracts showed the highest antioxidant and antimicrobial activities. The studied individual components obtained from chitosan-treated grapevines could represent an added value due to the increased antioxidant and antibacterial potentials. The phenolic compounds found in components may be used in food and pharmaceutical industries as natural food preservers and antibiotic adjuvants.

## 1. Introduction

Wine production is one of the best-known trades and tourism sectors in Portugal. The origin of wine production is unknown, but it is recognized as one of the oldest drinks, believed to be older than writing itself [1]. Wine production in Portugal has a huge economic, environmental, technological and social impact, and its production exceeds 7 million hectoliters according to the National Statistics Institute (INE) [2]. Several different wines are produced in Portugal, differentiated mainly by the region of vine growing. There is wine production throughout the country; however, the vineyards of the Douro Demarcated Region in Northern Portugal are the most recognized internationally. The Sousão variety (also called Vinhão) is one of the main red grapes varieties found in the Douro Demarcated Region. This is a late maturing variety and is not considered a truly teinturier variety due to its pink flesh; nevertheless, the main feature of this variety is its color, giving rise to dark wines with a very strong color [3,4]. Sousão is highly appreciated in the composition of Port wine, giving it color and acidity, essential conditions for fortifications intended for aging. In wine production there are some by-products resulting from winemaking that are considered waste since they are not of interest in the winemaking process. These by-products are mainly composed by discarded grape peels, seeds, stems, leaves and sludge, and constitute approximately 20% of the grape weighed for winemaking [5,6]. Nevertheless, such by-products are still rich in bioactive compounds, such as phenolic acids, stilbenes and flavonoids, including flavan-3-ols, flavonols, a stilbene and anthocyanins [6]. Therefore, they have the potential to be used in many other applications with possible commercial use, such as food additives and/or nutraceuticals [7]. Phenolic compounds are secondary metabolites synthesized by various species of plants and fungi, which protect against UV light, insects, viruses and bacteria. They may also be used by some plants as growth inhibitors of other competing plants. Widely distributed throughout the plant kingdom, phenolic compounds are considered important constituents in food for their contribution to taste, color and nutritional properties [8]. Phenolic compounds have been shown to have high antioxidant capacity, which is responsible for preserving the taste and color of foods, preventing the loss of vitamins and oxidative damage in living systems, and exhibit several physiological activities such as anticarcinogenic, anti-inflammatory, antiallergic, antihypertensive, and antimicrobial activities [9]. The increasing prevalence of antibiotic-resistant bacteria is one of the greatest medical challenges of our time. Therefore, there is a need to develop new approaches to tackle antimicrobial resistance. The antimicrobial spectrum of phenolic compounds is very broad, being not only active against bacteria, but also against plant and human pathogenic fungi [10,11,12]. Polyphenols can act on different cell types and through different mechanisms, e.g., rupture of the outer cell membrane, complexation with the cell wall, substrate deprivation, interacting with genetic material, enzymatic inactivation, altering the structure and function of the cytoplasmic membrane, disrupting proton and electron flow and inhibiting active transport [10,13]. Despite all these mechanisms of action, it is believed that the main and most common mechanism of antimicrobial actions of phenolic compounds is through interactions with the cell membrane [14].

Chitosan is a natural biopolymer, derived from arthropods exoskeleton and cell wall of fungi and carry highly favorable biological properties (biodegradability, biocompatibility, non-allergenicity and one of the most abundant renewable carbon source) that make it valuable to sustainable and improved agricultural and industrial practices [15,16]. The elicitor activity of this natural biopolymer arises from interaction of its polycationic molecule with negatively charged phospholipids of host cell membranes [17]. Chitosan is an ideal protective coating for fruits and vegetables because it has a disease-suppressive effect resulting from physical and biochemical mechanisms. That is why application of chitosan treatment is considered a suitable alternative to control many pre- and postharvest grapevine diseases, such as gray mold and powdery mildew, and prolongs storage life and controls decay of fruits [17,18]. According to Cho et al. (2008), treatment with this polysaccharide has been shown to stimulate plant growth, as well as to increase the content of phenolic compounds and antioxidant power [19,20]. Recently we performed chitosan application on two different grape varieties (cv. Touriga Franca and cv. Tinto Cao) and total phenolics/total anthocyanins/total tannins were analyzed; additionally ROS pathway genes were observed to be increasingly expressed upon application, and correlation could be drawn [16,21]. In opposition, Portu et al. (2016) did not find a substantial impact on the phenolic composition of grapes and wines following chitosan treatment [22]. This could be related to different grapevine varieties, thus requiring additional study. Moreover, when chitosan is used in the form of nanoparticles, its properties can be enhanced. These nanoparticles have been described as being a material with improved physical and biochemical properties and according to Ferrão et al. (2018) act as a more effective antimicrobial than chitosan gel, due to their high surface area and charged density, which interact with the surfaces of bacterial cells [23]. Up until now, reports regarding in-field investigations of chitosan applications on grapevine plants are still scarce. Therefore, in this study, the in-field application of chitosan nanoparticles was compared with chitosan solution treatment and with a control (without treatment) using a grapevine cultivar relevant for Douro wine production, but has so far not been studied. Individual phenolic compounds and respective activity against pathogenic bacteria were also evaluated, as they had not been assessed in previous studies and may be of interest, considering that those individual components will later be present in winemaking by-products with potential application in pharmaceutical/nutraceutical industries. Thus, in the present study, phenolic compounds were extracted from grape skin, seeds and stems from the “Sousão” variety treated and not treated with chitosan and evaluated for their antioxidant activity and antibacterial properties against antibiotic resistant bacterial strains.

## 2. Materials and Methods 

### 2.1. Chitosan

Chitosan (fungal origin) was purchased from Kitozyme (Belgium) and dissolved in 0.01% acetic acid solution by concentration of 0.01% chitosan (*w*/*v*) (76 kDa molecular wt and 85% deacetylation degree). For the production of chitosan nanoparticles, chitosan solution was prepared (0.5% *w*/*v*) in 0.01 M aqueous acetic acid and pH was raised to 4.8 with 10N NaOH. Aqueous tripolyphosphate solution (0.25%, *w*/*v*) was mixed with chitosan solution drop by drop continuously under magnetic stirring (500 rpm) at room temperature with a ratio of 3:1 (chitosan: tripolyphosphate) until the solution started turning to milky appearance [24]. Nanoparticles were centrifuged at 10,000 rpm/20 min and supernatant was discarded, pellet was rinsed with distill water and dissolved.

### 2.2. Plant Material and Extracts

Nine marked grapevine lines of Sousão variety with 20 plants each were selected on the basis of phenotypic similarity for the experimental setup. Three grapevine lines were used as control, and leaves and berries of six grapevine plants of selected lines were sprayed with chitosan solution (three grapevine lines) and chitosan nanoparticles (three grapevine lines) at the beginning of veraison and at complete veraison stage. A total of two applications of the treatment using chitosan solution (0.01%) and chitosan nanoparticles (0.001%) were performed and samples were collected at final maturity of berries; interval time between the two treatments was 16 days. The present study was conducted in terraced vineyard garden of Casa da Mateus (41°19′ N, 7°44′ W, 500 m above mean sea level), Baixo Corgo sub-region of the Demarcated Douro Region, Vila Real, northern Portugal. Plot had typical grass covered morainic, loamy sand with 15% gravel and developed according to Guyot system. Average temperature, humidity, and wind velocity were recorded as 23.4 °C, 57.4%, and 6.5 km/h, respectively, during the application until sample collection. Fifty berries were randomly collected for analysis of seeds and skins (was separated manually) from each treatment as well as control and frozen immediately followed by freeze drying. Stem tissues were collected from the wine making process, where stems were separated from grape bunch before undergoing to crushing and fermentation. Grape individual components were freeze-dried, mill-powdered and stored in a desiccator.

The extraction of phenolic compounds from winemaking by-products of the Sousão variety was performed using water/ethanol (50:50) mixture as previously described [6]. Briefly, two grams of lyophilized plant material (skin, seeds and stems) were weighted and extracted in 100 mL of solvent. Samples were incubated for 2 h with constant stirring followed by sonication during 5 min. Extracts were centrifuged at 10,000 rpm for 15 min at 4 °C and the pellet was re-extracted. Supernatants were filtered through (polytetrafluoroethylene) PTFE 0.2 µm and the solvents were evaporated under vacuum on rotary evaporator at 40 °C. The dry residues obtained were dissolved in DMSO to a final concentration of 100 µg/mL. Extraction was performed in duplicate for each sample.

### 2.3. Determination of Total Phenolic and Anthocyanin Contents

The determination of total content of phenols and anthocyanins in the extracts of the stems, seeds and peels was performed spectrophotometrically as previously described [25]. A mixture of 200 µL of the extracts and 3.8 mL of HCl 1.0 M was incubated at room temperature for 3 h. To determine the anthocyanin content in samples, the absorbance of the mixture was measured at 520 nm and for the total phenol content the absorbance was measured at 280 nm. The reference solution used was DMSO and quartz crystal cuvettes were used for every experiment. Each assay was done in triplicate and results expressed as mean ± standard deviation (SD).

### 2.4. Determination of Tannin Content

For the determination of total tannin content it was used the method described by Sarneckis et al. (2002) with some modifications [26]. For the treatment samples, 600 µL methyl cellulose solution (0.04%) (prepared according to the manufacturer’s instructions) was added to 50 µL of each sample in a 2 mL centrifuge tubes and the solution was mixed by tube inversion. The tubes were incubated at room temperature for 3 min. Four hundred microliters of saturated ammonium sulphate solution were added, and the tubes were filed with water to make up to 2 mL. Finally, the mixture was incubated at room temperature for 10 min and then centrifuged for 15 min at 10,000× *g*. The absorbance was measured at 280 nm. For the control samples, water was used instead of methyl cellulose solution. Four hundred microliters of saturated ammonium sulphate solution were added to 30 µL of each sample and volume made up with water. The tubes were inverted several times, incubated at room temperature for 10 min and centrifuged for 15 min at 10,000× *g*. The absorbance was measured at 280 nm.

### 2.5. HPLC-DAD Analysis

Polyphenols identification and quantification was done using a HPLC-DAD. A HPLC (Gilson) system equipped with one mixture chamber (Gilson, model 811A), two pumps (Gilson, model 305 and 306), automatic injector (Gilson, model 231X), oven (Jones chromatography) and a diode array detector (DAD) (Thermo, Finnigan Surveyor detector) was used to identify the polyphenols present in the different parts of the grapes. The mobile phase was composed by water with 0.1% of TFA (solvent A) and acetonitrile with 0.1% TFA (solvent B). 10 µL of each extract were injected into a C18 column (250 mm × 4.6 mm; 5 μm particle size, ACE, Advanced Chromatography Technologies, Aberdeen, United Kingdom). The elution was performed at a flow rate of 1 mL/min with the following gradient: 0 min 100% A, 5 min 100% A, 15 min 80% A, 30 min 50% A, 45 min 0% A, 50 min 0% A, 55 min 100% A, and 60 min 100% A.

Catechins, phenolic acids, flavonoids and anthocyanins were quantified at 280, 320, 370 and 520 nm, respectively. Compounds were identified through peak retention time, UV spectra and UV maxima absorbance bands as well as by comparison with commercial internal standards. Quantification was accomplished using commercial standards of the identified compounds. All chemicals used in this study were of analytical grade. Naringin was purchased from Sigma-Aldrich (Tauferkichen, Germany). Gallic acid, protocatechuic acid, (+)-catechin, (−)-epicatechin, (−)-epigallocatechin, procyanidin B2, resveratrol, chlorogenic acid, coumaric acid, caffeic acid, rutin, cyanidin-3-*O*-glucoside, delphinidin-3-*O*-glucoside malvidin-3-*O*-glucoside, petunidin-3-*O*-glucoside standards were purchased from Extrasynthèse (Genay, France). Methanol and acetonitrile were HPLC gradient and purchased from Thermo Fisher.

### 2.6. Determination of Antioxidant Activity

The antioxidant activity was determined by DPPH (2,2-diphenyl-1-picrylhydrazyl) and reducing power assays.

For the scavenging activity assay, from each of the different extract solutions (concentration range from 1 mg/mL to 0.03 mg/mL for seeds and peels, and from 2.5 mg/mL to 0.06 mg/mL for the stem) 30 μL were used and added to 270 μL of a methanolic solution containing DPPH radicals (6 × 10^−5^ M). A control was prepared with extraction solvent instead of the extract solution for later comparison. The mixture was protected from light and after 60 min the decrease of the DPPH radical was determined using an Epoch™ 2 Biotek Microplate Reader (BioTek Instruments, Inc., Winooski, VT, USA) at a wavelength of 517 nm. Radical scavenging activity (RSA) was calculated by the percentage of discoloration of the DPPH solution using the following equation:

$$\%\mathrm{RSA}=\left[\frac{A_{\mathrm{DPPH}}-A_{\mathrm{sol}}}{A_{\mathrm{DPPH}}}\right]\times 100$$

where A_sol_ corresponds to the absorbance of the DPPH solution in the presence of different extract concentrations and A_DPPH_ corresponds to the absorbance of the control. The extract concentration corresponding to 50% of the radical scavenging activity (EC_50_) was calculated by interpolation from the percentage RSA plot as a function of the extract concentration.

The reducing power of the extracts was determined according to the method described by Miceli et al. (2009), with slight modifications [27]. For each of the different dilutions of the extracts (250 μL) was added 250 µL of sodium phosphate buffer (0.2 M at pH = 6.6) and 250 µL of 1% potassium ferricyanide solution. The mixture was incubated at 50 °C for 20 min and, after cooling, 250 µL of trichloroacetic acid (10%) was added. After centrifugation, 0.5 mL of the supernatant was removed and 0.5 mL of deionized water and 0.2 mL of FeCl_3_ (0.1%) were added. A blank was further prepared using extraction solvent instead of extract. The absorbance of the mixtures was measured at 690 nm using an Epoch™ 2 Biotek Microplate Reader (BioTek Instruments, Inc., USA). The extract concentration corresponding to the EC_50_ was calculated from the graph obtained by plotting the absorbance at 690 nm as a function of the corresponding extract concentration. For both assays (DPPH and reducing power) Trolox was used as positive control.

### 2.7. Bacterial Isolates

Antimicrobial susceptibility testing was performed against four multidrug resistant Gram-positive bacteria: *Enterococcus faecalis* vanB2-C3735 [28], *Enterococcus faecium* vanA-C2302 [29], *Staphylococcus aureus* C5932 (MRSA CC398) [30], *Staphylococcus epidermidis* C3658 (linezo-R) [31], and 4 multiresisatnt Gram-negative bacteria: *Salmonella enteritidis* C4220, *Escherichia coli* C999 (CTX-M-15) [32] *Klebsiella pneumoniae* C1370 (CTX-M-15) [32], *Pseudomonas aeruginosa* C4660 (VIM-2) [33]; and two Gram-positive foodborne strains *Listeria monocytogenes* ATCC700302 and *Bacillus cereus* ATCC1306. The strains are part of the University of Trás-os-Montes and Alto Douro and University of La Rioja collections. All the bacterial strains were subcultured from the original culture in Brain Heart Infusion (BHI) agar (Oxoid, UK) for 24 h at 37 °C. Müller-Hinton (MH) agar (Oxoid, UK) was used for the antimicrobial susceptibility assay. All the bacterial strains were subculture from the original culture in Brain Heart Infusion (BHI) agar (Oxoid, UK) for 24 h at 37 °C. Müller-Hinton (MH) agar (Oxoid, UK) was used for the antimicrobial susceptibility assay.

### 2.8. Antimicrobial Susceptibility Test

The antimicrobial susceptibility assay was performed using Kirby-Bauer disc diffusion method. The measurement of bacterial growth inhibition was carried out as previously described [6]. Each bacterial strain was seeded in BHI agar plates and incubated overnight at 37 °C. A few colonies were suspended in physiological solution to a turbidity equivalent to 0.5 McFarland standard and 100 µL was plated onto MH plates. The initial extract solution of 100 µg/mL was diluted with DMSO to 75, 50, 25 and 10 µg/mL. Twenty microliters of each extract concentration were loaded on sterile blank discs (6 mm diameter) and the discs were placed onto inoculated MH plates. The plates were incubated for 24 h at 37 °C. The inhibition zones were measured with a ruler, recorded and considered as indication for antibacterial activity. Discs loaded with DMSO were used as negative control and antibiotic discs were used as positive control. The test was performed in duplicate.

### 2.9. Statistical Analysis

The results were expressed as mean values and standard deviation (SD). All results were analyzed using IBM SPSS Statistics for Mac, Version 26.0. (IBM Corp., Armonk, New York, NY, USA). One-way analysis of variance (ANOVA) followed by Tukey’s HSD Test with *p* = 0.05 was performed. To verify the homogeneity of variances, Levene’s was implemented to verify the homogeneity of variances. For the individual phenolic compounds’ quantification, a Student’s t-test was used to determine the significant difference, with *p* = 0.05.

## 3. Results and Discussion

### 3.1. Phenolic Profile Analysis

In this study, Sousão vines were treated with a chitosan solution and chitosan nanoparticles in order to investigate the effect of these treatments in phenolic compounds, and their consequent influence in the antioxidant and antibacterial activities. Previous studies have investigated the effect of chitosan on the phenolics of grape pomace and wine; however, as far as we know, this is the first report on the chitosan treatment effect on the individual components of grapes: Skins, seeds and stems.

Table 1 shows the total phenolic content (TPC), total anthocyanin content (TAC) and total tannin content (TTC) of the skins, seeds and stems of Sousão variety grapes with no treatment (control), treated with a chitosan solution and treated with chitosan nanoparticles. Regarding the control group, skins showed a higher TPC, followed by seeds and stems extracts. In contrast, seeds showed a much higher tannin content than the skins or stems extracts. Similar results were obtained in previous studies carried out on other different grape varieties, namely Merlot, Touriga Nacional and Preto Martinho, where the TTC was also highest in the seeds, whereas the stems presented the lowest tannin content [6,34]. Nevertheless, due to the small proportion of this component in the cluster, stem tannins have less importance [34]. The treatment with chitosan seems to have influenced the phenolic content of grape components. There was an increase in the TPC of skins and stems after the vines treatment with chitosan solution, as well as the TAC in the skins. Nevertheless, the TPC increase was only statistically significant in stems extracts. According to the obtained results, the chitosan solution had a negative effect on the TTC leading to a decrease in tannin content in all grape components, despite being only significant in the skins. In general, the grapes treated with chitosan nanoparticles did not presented better results compared to the treatment with chitosan solution, with TPC and TTC decrease compared to control being the most striking result for the skins extracts. Nevertheless, in grapes treated with nanoparticles, TAC remained similar to that of the non-treated grapes. Regarding the TTC of the stems, those treated with chitosan nanoparticles presented the highest value. Only a few studies have investigated the chitosan effect on the phenolic composition of grapes and reported that chitosan treatment did not have a considerable effect on the phenolic content [22,35]. Accumulation and increasing of the TPC after chitosan treatment had also been reported for other vegetables such as spinach [36]. In this study, despite an increase of TAC observed in both groups treated with chitosan (solution or nanoparticeles), the chitosan application did not influence notably the anthocyanin content in grape skins in comparison to the control since the observed difference were not statically significant. Duxbury et al. (2004) investigated the effect of chitosan treatment on Cabernet Sauvignon vines and reported that this treatment had no effect on the total phenolic and anthocyanin contents of grapes when compared to the control [35]. Nevertheless, Ferri et al. 2009 reported that chitosan treatment may lead to an accumulation of anthocyanins [37]. Despite the fact that the influence of chitosan had not been studied in detail and is still not completely understood, there are evidence that chitosan treatment may activate key enzymes of the phenylpropanoid pathway, in particular phenylalanine ammonia lyase which is the key enzyme that catalyzes the first step in the phenolic biosynthesis [22]. Chitosan is used to control many grapevine diseases, and its formulations in concentrations like the one used in our study showed a decrease of the incidence of grapevine infections. Additionally, Iriti et al. (2011) found that chitosan application improved TPC of grapes when compared with conventional fungicides [18].

The individual phenolic compounds present in grapes components were determined by reverse phase HPLC-DAD. Identification of the compounds was accomplished by comparison with standards and literature and the quantification was archived by calibration curves of external standards and is expressed in epicatechin equivalents. The polyphenolic profiling revealed a total 22 polyphenols (Table 2).

Seed extracts were mainly constituted by gallic acid and flavanols. The most abundant compound in seeds was epicatechin gallate which had similar concentrations in control (3.01 ± 0.35 µg/mg) and chitosan treated grapes (3.07 ± 0.22 µg/mg), while in grapes treated with chitosan nanoparticles the concentration decreased significantly. Epicatechin units along with catechin, epicatechin gallate, and epigallocatechin are the building blocks of the proanthocyanidins which are a type of condensed tannin [38]. Chitosan solution promoted a minor increase of flavanols when compared to the control group. Nevertheless, gallic acid was present in a slightly higher, but not significative, concentration in grape components treated with chitosan nanoparticles. Therefore, chitosan treatment seems to have an effect, though small, on the content of proanthocyanidins in seeds extracts. These compounds have a major role in wine quality and can be incorporated into the wine during the maceration of seeds. Regarding stems extracts, 17 phenolic compounds were identified. Surprisingly, the two most abundant compounds in stems were two anthocyanins, malvidin-3-glucoside and peonidin-3-glucoside. Their concentration increased significantly in stems treated with chitosan (16.07 ± 0.74 and 18.12 ± 0.69 µg/mg, respectively) when compared with the control grapes (11.29 ± 0.33 and 13.30 ± 0.69 µg/mg, respectively). Nevertheless, the treatment with chitosan nanoparticles had a negative effect on the concentration of these anthocyanins. Similar to malvidin-3-glucoside and peonidin-3-glucoside, catechin (the third most abundant compound present in stems) also significantly increased in concentration in chitosan treated vines. In contrast to our results, Romanazzi et al. (2006) studied the influence of chitosan treatment on catechin concentration and reported that chitosan did not increase the catechin concentration in grapes [17]. Quercetin-3-*O*-rutinoside, also known as rutin, is an important phenolic compound with proved beneficial effects on human health. These compounds were present in stems; however, chitosan had no effect on their concentration. Studies have suggested that the application of elicitors, as chitosan, exerts an impact on flavonol synthesis. Flavonols and anthocyanins share a big part of their metabolic pathway; however, the anthocyanin biosynthesis is preferentially activated by chitosan when compared to the biosynthesis of flavonols [22,39]. Phenolic acids, such as *p*-coumaric acid and ferulic acid, are usually present in stems as they constitute the lignin which is a key component of stems [40]. In our study, ferulic and *p*-coumaric acids were detected in stems in low concentrations being both increased in concentration in stems treated with chitosan; yet, only the concentration of ferulic acid was significantly increased. Concerning the skins, as expected, malvidin-3-*O*-glucoside and peonidin-3-*O*-glucoside were the most abundant compounds, followed by delphinidin-3-*O*-glucoside. The concentration of malvidin-3-*O*-glucoside remained unaltered in skins of grapes treated with chitosan solution and nanoparticles relative to control. As for the concentration of peonidin-3-*O*-glucoside in relation to the control grapes, it decreased in grapes treated with chitosan solution and increased in grapes treated with nanoparticles. Malvidin-3-glucoside is the most abundant anthocyanin in nearly all grape varieties while grape content on other anthocyanins differs between different grape varieties. Malvidin-3-glucoside and peonidin-3-glucoside are the final products of anthocyanin pathway biosynthesis and, therefore, this probably influence the increasing of concentration of malvidin-3-glucoside and peonidin-3-glucoside during ripening.

### 3.2. Antioxidant Activity

The antioxidant activity of each grape component of vines with different treatments was determined by DPPH and reducing power assays. Results from both assays are expressed in effective concentration (EC50), and the lower the value the higher is the antioxidant power. The results obtained are shown in Table 3, and according to it, seed extracts had the higher antioxidant activity in all groups tested and in both assays. Regarding the control and the chitosan treated grapes, stems presented a higher antioxidant power than the skins using the DPPH assay; however, the opposite happened using the reducing power assay. Skins had the highest content in phenolic compounds, followed by seeds and stems. Nevertheless, seeds presented a higher antioxidant power which may be due to their elevated content in tannins and proanthocyanidins. Regarding the effect of chitosan treatment in the antioxidant power of the grape components, the majority of the results are in accordance with the obtained in the TTC. The seeds’ antioxidant power remains unaltered with the treatments in the DPPH assay while the results of the reducing power assay show that both chitosan treatments results in an increase in the antioxidant activity being more accentuated in the seeds of grapes treated with chitosan solution. As for the stems, the DPPH results of the control group and the group treated with chitosan nanoparticles are quite similar whereas the antioxidant activity improve in stems from grapes treated with chitosan solution. Nevertheless, in the reducing power assay, stems present a higher antioxidant power from grapes treated with nanoparticles. Skins presented the poorest results for both assays; nevertheless, the chitosan treatment improved the antioxidant results. Singh (2016) applied chitosan solution at different concentrations 0.1, 0.05, 0.01, 0.005, 0.001 mg/mL on spinach leaf surface and reported that the maximum increase in the antioxidant activity was achieved with the chitosan concentration at 0.01 mg/mL which is the same concentration used in our study [36]. Furthermore, the author showed a correlation between the content in phenolic compounds and the antioxidant activity. In our study, we observed that chitosan treatment improved antioxidant activity in comparison with the control group. Some studies have reported similar results [20], while others have reported that chitosan treatment improved the antioxidant power when compared with the results of other elicitors but not with respect to untreated grapes [18]. When elicitors, such as chitosan, are applied to a plant, it generates reactive oxygen species (ROS) as a defense response resulting in the induction of oxidative stress. The production of ROS stimulates the synthesis of defensive compounds, such as phenolic compounds [36,41].

### 3.3. Antimicrobial Activity

The antimicrobial activity of chitosan to prevent plant infection by plant pathogens is well known. However, there are no studies reporting the effect of chitosan on the antibacterial properties of plant extracts against multidrug-resistant bacteria. The assessment of antimicrobial activity by grape components treated with chitosan was performed using the Kirby-Bauer disc diffusion method. The results of antibacterial activity are shown in Table 4. Gram-positive bacteria presented a higher susceptibility to extracts than Gram-negative bacteria. These results are independent of chitosan treatment since other studies used extracts of grape individual components and have reported similar results. Despite the fact that all Gram-negative bacteria used in this study present multiple antibiotic resistances, it is known that cell walls of Gram-negative represent a major barrier for the entry of phenolic compounds into cell cytoplasm due to the repulsion between lipopolysaccharide found in the surfaces of Gram-negative bacteria and phenols [42,43]. In this study, phenolic extracts did not have any effect against *E. coli*, *K. pneumoniae* and *S. enteritidis*. The resistance of phenolic compounds action by multidrug-resistant bacteria is not completely understood. *P. aeruginosa* was the only Gram-negative bacteria showing susceptibility to almost all extracts used in this study with better results for the seeds extracts (MIC of 25 µg/mL). *P. aeruginosa* presented several resistances to antibiotics, in particular, to carbapenems antibiotics, nevertheless, it was not resistance to antibiotics which in the mechanism of resistance are efflux pumps as it happened in the other Gram-negative bacteria tested.

In general, seed extracts had the better antibacterial efficacy, since they suppress the growth of bacteria at low concentrations, followed by the stems and the seeds. These results are in accordance with the obtained in antioxidant activity. Phenolic antioxidants are known to inhibit the growth of bacteria [44]. Nevertheless, studies have shown a correlation between the TPC and the antimicrobial activity of polyphenols extracted from winery by-products, being the highest TPC associated with the better results of antimicrobial activity [6,45,46]. Even though, seeds inhibited the growth of 5 out of 10 bacteria tested there was no significant difference between the control and the chitosan treated grapes. Stems extracts of control grapes had effect on 3 strains while extracts of stems treated with chitosan solution and nanoparticles presented better results inhibiting the growth of five and six bacterial strains, respectively; however, strains were inhibited at high concentrations. As for the skin extracts, they had a moderate efficacy, since the control and the grapes treated with chitosan solution inhibited the growth of 6 out of 10 bacterial strains. However, skins treated with chitosan nanoparticles had an inhibitory effect on only one strain (*S. epidermidis*), which may reflect the decrease of TPC and TTC in skins extracts after chitosan treatment. *E. faecalis* and *E. faecium* share the genus *Enterococcus*; however, there were quite different results of antimicrobial activity. *E. faecalis* was inhibited by almost all extracts whereas the growth of *E. faecium* was not suppressed by any of the used extracts. Both strains are resistant to vancomycin and share similar antibiotic resistances and resistance genes, the only significant difference is the fact that *E. faecalis* harbors the *van*B2 gene and *E. faecium* harbors the *van*A (both conferring resistance to vancomycin). Similar results were obtained in our previous study, as *E. faecalis* was inhibited by almost all grape extracts and, although *E. faecium* was inhibited by some extracts, it was present in high concentrations [6]. *L. monocytogenes* and *B. cereus* are usually associated with food-borne infections. All extracts at moderate concentrations, except skins treated with nanoparticles, were able to suppress the growth of *L. monocytogenes*. As for *B. cereus*, extracts of grape components treated with chitosan solution were had higher effect on inhibiting the growth of this strain. The extracts, in particular those extracted from grape components treated with chitosan, were effective against foodborne bacteria which shows that plants treated with chitosan may be a potential source of phenolics which can be used as food preservers. Chitosan elicits the plant defense system against a broad spectrum of pathogens. Chitosan application results in the accumulation of phenolic compounds in treated plant tissues due to the stimulation of the biosynthetic route that leads to polyphenol synthesis [20]. However, the antimicrobial mechanisms of by-product extracts are still unclear, since there are hundreds of different bioactive compounds that can differ among different extractions methods.

## 4. Conclusions

The present study suggests that the use of chitosan as elicitor in grapevines, in addition to preventing plant infections, also increases the phenolic and tannin contents. However, the application of chitosan nanoparticles in grapevines did not present the results expected as grape components treated with nanoparticles did not present higher antioxidant nor antibacterial activities in comparison to the treatment with chitosan solution. Furthermore, chitosan-treated grape components showed a higher antioxidant and antibacterial activities than non-treated grapes. Chitosan treatment caused a potent antibacterial activity in the winery by-products: stems, seeds and skins of grapevines against different important multidrug-resistance and food-borne bacteria which is noteworthy. As winery industries produce a substantial amount of by-products, it is becoming more imperative to solve this problem that can cause environmental issues like pollution. It is necessary to develop systems and different applications for the use of these winery waste treatment.

## Figures and Tables

**Table 1:** Total phenolic content (TPC), total anthocyanin content (TAC) and total tannin content (TTC) of the skins, seeds and stems of Sousão variety with no treatment (control), treated with chitosan and with chitosan nanoparticles (mean ± SD, n = 3).

Grape Component		TPC (µg/mg) ^1^	TAC (µg/mg) ^2^	TTC (µg/mg) ^1^
Skins	Control	52.519 ± 2.52 ^a^	11.21 ± 0.44 ^a^	16.16 ± 0.87 ^a^
	Chitosan	53.39 ± 1.03 ^a^	12.25 ± 0.23 ^a^	11.22 ± 0.49 ^b^
	Chitosan nanoparticles	45.652 ± 3.74 ^a^	11.51 ± 0.52 ^a^	7.91 ± 0.42 ^c^
Seeds	Control	42.36 ± 0.28 ^a^	n.d.	38.46 ± 1.02 ^a^
	Chitosan	41.30 ± 0.40 ^a^	n.d.	36.07 ± 2.34 ^a^
	Chitosan nanoparticles	42.064 ± 1.01 ^a^	n.d.	38.57 ± 2.64 ^a^
Stems	Control	22.12 ± 0.40 ^a^	n.d.	10.37 ± 0.43 ^a^
	Chitosan	24.93 ± 0.61 ^b^	n.d.	8.66 ± 1.56 ^a^
	Chitosan nanoparticles	22.60 ± 0.27 ^a^	n.d.	13.71 ± 0.22 ^b^

n.d.: not determined. For each group an ANOVA analysis was performed, with different letters indicating significant differences (*p* < 0.05). ^1^ Values expressed as mg of epicatechin equivalents/g of residue. Different letters indicate significant differences (*p* < 0.05). ^2^ Values expressed as mg of malvidin-3-glucoside equivalents/g of residue. Different letters indicate significant differences (*p* < 0.05).

**Table 2:** Polyphenolic compounds (µg/mg of residue) found in grape skins, seeds and stems of Sousão variety with no treatment (control), treated with chitosan and with chitosan nanoparticles (mean ± SD, n = 3).

	**SEEDS**			
	**λ máx (nm)**	**Control**	**Chitosan**	**Chitosan Nanoparticles**
Gallic acid	280	0.32 ± 0.02 ^a^	0.34 ± 0.03 ^a^	0.37 ± 0.07 ^a^
Catechin	280	0.62 ± 0.07 ^a^	0.93 ± 0.17 ^a^	0.84 ± 0.19 ^a^
Epicatechin	280	0.72 ± 0.05 ^a^	0.84 ± 0.19 ^a^	0.71 ± 0.09 ^a^
Epicatechin gallate	280	3.01 ± 0.35 ^a^	3.07 ± 0.22 ^a^	1.07 ± 0.04 ^b^
	**STEMS**			
	**λ máx (nm)**	**Control**	**Chitosan**	**Chitosan Nanoparticles**
Gallic acid	280	0.37 ± 0.03 ^a^	0.24 ± 0.01 ^b^	0.24 ± 0.02 ^b^
*o*-Coumaric acid	320	0.39 ± 0.02 ^a^	0.21 ± 0.01 ^b^	0.25 ± 0.05 ^b^
Catechin	280	2.37 ± 0.15 ^a^	5.16 ± 0.29 ^b^	2.24 ± 0.12 ^a^
Chlorogenic acid	320	2.45 ± 0.55 ^a^	3.58 ± 0.16 ^b^	2.08 ± 0.08 ^a^
Epicatechin	280	0.41 ± 0.03 ^a^	0.79 ± 0.07 ^b^	0.66 ± 0.06 ^b^
Cyanidin	520	0.48 ± 0.04 ^a^	1.42 ± 0.04 ^b^	0.76 ± 0.04 ^c^
Malvidin	520	1.30 ± 0.12 ^a^	2.97 ± 0.17 ^b^	1.95 ± 0.05 ^c^
*m*-Coumaric acid	320	0.51 ± 0.02 ^a^	1.05 ± 0.04 ^b^	0.44 ± 0.12 ^a^
Gallocatechin gallate	280	0.34 ± 0.10 ^a^	n.d.	0.06 ± 0.01 ^b^
Malvidin-3-glucoside	520	11.29 ± 0.33 ^a^	16.07 ± 0.74 ^b^	11.75 ± 0.12 ^a^
Epicatechin gallate	280	1.91 ± 0.12 ^a^	1.94 ± 0.06 ^a^	1.85 ± 0.09 ^a^
Quercetin-3-*O*-rutinoside	370	1.28 ± 0.05 ^a^	1.19 ± 0.04 ^a^	0.9 ± 0.04 ^b^
Petunidin	520	1.24 ± 0.12 ^a^	1.58 ± 0.10 ^b^	1.13 ± 0.1 ^a^
Peonidin-3-glucoside	520	13.30 ± 0.69 ^a^	18.12 ± 0.69 ^b^	11.89 ± 0.14 ^c^
*p-*coumaric acid	320	0.31 ± 0.08 ^a^	0.46 ± 0.02 ^a^	0.44 ± 0.13 ^a^
Catechin gallate	280	0.54 ± 0.12 ^a^	0.22 ± 0.06 ^b^	0.25 ± 0.04 ^b^
Ferulic acid	320	0.15± 0.02 ^a^	1.14 ± 0.05 ^b^	1.39 ± 0.11 ^c^
	**SKINS**			
	**λ máx (nm)**	**Control**	**Chitosan**	**Chitosan Nanoparticles**
Delphinidin-3-*O*-glucoside	520	40.12 ± 2.84 ^a^	43.48 ± 1.66 ^a^	40.31 ± 0.16 ^a^
Cianidina-3-*O*-glucoside	520	1.03 ± 0.07 ^a^	1.13 ± 0.20 ^a^	0.94 ± 0.06 ^a^
Malvidin-3-*O*-glucoside	520	75.40 ± 4.92 ^a^	75.71 ± 8.20 ^a^	75.45 ± 11.27 ^a^
Peonidin-3-*O*-glucoside	520	70.09 ± 1.20 ^a^	67.25 ± 1.62 ^a^	76.23 ± 0.11 ^b^
Delphinidin-3-*p*-coumarylglucoside	520	0.94 ± 0.18 ^a^	0.93 ± 0.27 ^a^	1.15 ± 0.28 ^a^
Peonidin-3-acetylglucoside	520	8.15 ± 0.53 ^a^	6.71 ± 0.04 ^a^	9.06 ± 1.15 ^a^
Malvidin-3-acetylglucoside	520	1.79 ± 0.05 ^a^	1.52 ± 0.07 ^a^	2.02 ± 0.34 ^a^
Cyanidin-3-*p*-coumaryl glucoside	520	2.47 ± 0.12 ^a^	2.24 ± 0.23 ^a^	2.71 ± 0.11 ^a^
Quercetin-3-*O*-rutinoside	370	1.90 ±0.25 ^a^	2.12 ± 0.07 ^a^	2.42 ± 0.16 ^b^
Gallic acid	280	1.88 ± 0.10 ^a^	2.12 ± 0.04 ^a^	1.96 ± 0.01 ^a^

For total phenolic compounds an ANOVA analysis was performed, with different letters indicating significant differences (*p* < 0.05).

**Table 3:** Antioxidant activity of the skins, seeds and stems of Sousão variety with no treatment (control), treated with chitosan and with chitosan nanoparticles expressed in EC_50_ (mg/mL) (mean value ± SD, n = 3).

Grape Component		DPPH	Reducing Power
Skins	Control	0.368 ± 0.009 ^a^	1.416 ± 0.147 ^a^
	Chitosan	0.313 ± 0.003 ^b^	0.752 ± 0.036 ^b^
	Chitosan nanoparticles	0.455 ± 0.030 ^c^	0.854 ± 0.034 ^b^
Seeds	Control	0.059 ± 0.001 ^a^	0.450 ± 0.071 ^a^
	Chitosan	0.058 ± 0.001 ^a^	0.104 ± 0.003 ^b^
	Chitosan nanoparticles	0.057 ± 0.01 ^a^	0.120 ± 0.003 ^b^
Stems	Control	0.247 ± 0.008 ^a^	2.368 ± 0.035 ^a^
	Chitosan	0.178 ± 0.020 ^b^	0.913 ± 0.079 ^b^
	Chitosan nanoparticles	0.214 ± 0.002 ^c^	0.490 ± 0.008 ^c^

For each group an ANOVA analysis was performed, with different letters indicating significant differences (*p* < 0.05).

**Table 4:** Minimum inhibitory concentration (MIC) and the inhibition zones (mm) of grape skins, stems and seeds of Sousão variety with no treatment (control), treated with chitosan and with chitosan nanoparticles.

	MIC (mg/mL) (Inhibition Zones (mm))								
	Control			Chitosan			Chitosan Nanoparticles		
	Skins	Stems	Seeds	Skins	Stems	Seeds	Skins	Stems	Seeds
Gram-positive									
*S. epidermidis*	50 (17)	-	50 (15)	75 (17)	75 (14)	50 (15)	50 (14)	100 (14)	25 (15)
*S. aureus*	50 (12)	100 (11)	25 (14)	50 (13)	-	25 (15)	-	75 (12)	25 (13)
*E. faecalis*	50 (10)	-	75 (12)	50 (12)	100 (11)	25 (12)	-	100 (9)	50 (11)
*E. faecium*	-	-	-	-	-	-	-	-	-
*L. monocytogenes*	75 (13)	75 (13)	25 (16)	75 (13)	50 (13)	25 (17)	-	75 (14)	25 (14)
*B. cereus*	100 (10)	-	-	100 (11)	75 (13)	-	-	100 (12)	-
Gram-negative									
*K. pneumoniae*	-	-	-	-	-	-	-	-	-
*E. coli*	-	-	-	-	-	-	-	-	-
*P. aeruginosa*	75 (10)	100 (10)	25 (12)	75 (10)	100 (12)	25 (11)	-	100 (10)	25 (11)
*S. enteritidis*	-	-	-	-	-	-	-	-	-
